# Special Issue “Natural Products in Drug Discovery and Development: 2nd Edition”

**DOI:** 10.3390/ijms27209169

**Published:** 2026-10-16

**Authors:** Tian-Le Gao

**Affiliations:** Institute of Materia Medica, Chinese Academy of Medical Sciences & Peking Union Medical College, Beijing 100050, China; tianlegao@imm.ac.cn

## 1. Introduction

The Special Issue “Natural Products in Drug Discovery and Development” returns for a second edition at a time when nature-derived molecules continue to demonstrate their enduring value to medicine. Across nearly four decades, natural products and their derivatives have remained a disproportionately productive source of new chemical entities, providing scaffolds, lead compounds and pharmacological tools that synthetic chemistry alone would be unlikely to access [1]. This reflects a deeper compatibility: as products of biological selection, natural products are predisposed to engage the densely connected molecular networks of living systems.

The first edition of this Special Issue assembled studies on the isolation and characterization of active constituents, the pharmacological actions of individual compounds, innovative formulations and combinational therapies, and first articulated the place of negative-entropy (neg-entropy) mechanisms in natural-medicine research. The present edition is a direct continuation of that effort: where the first edition sketched the conceptual vocabulary, this one begins to populate it with disease-resolved, mechanistically explicit examples. This Editorial situates the collected work within the framework of head goose molecules (HGMs), biological entropy (bio-entropy) homeostasis and the drug cloud, and highlights how the studies reported here exemplify that framework.

## 2. From Single-Target Blockade to Neg-Entropy Homeostasis

Modern pharmacology has long operated under the “one gene, one target, one drug” paradigm. The limitations of strict monotherapy are threefold. First, single-target drugs often improve a phenotypic marker only transiently, without correcting the molecular, cellular and tissue-level disorganization from which the marker arises. Second, biological networks are highly interconnected; blocking one pathway can drive the compensatory upregulation of parallel routes, producing acquired resistance—an effect documented for inhibitors as different as epigenetic readers and genotoxic chemotherapeutics. Third, excessive suppression of a physiological pathway can itself disturb homeostasis, provoking the metabolic and immune side effects that complicate long-term therapy. These limitations have motivated sustained interest in multi-target ligands and network pharmacology, which explicitly models drugs as modulators of interconnected protein networks rather than isolated keys for isolated locks [2,3].

A thermodynamic formulation of the same problem provides additional clarity. The second law dictates that an isolated system evolves spontaneously toward increasing entropy and disorder. A living organism, by contrast, is an open, dissipative system that continuously exchanges matter, energy and information with its environment, importing neg-entropy to offset its intrinsic tendency toward disorder [4,5]. Biological entropy can therefore be regarded as an extension of thermodynamic entropy into life, quantifying disorganization across molecular modifications, cellular function, organ physiology and whole-body regulation; rising entropy corresponds to structural damage, loss of physiological order and, ultimately, to disease and aging [5].

Under healthy conditions, five intrinsic neg-entropy capacities act in concert to resist this drift toward disorder: (i) metabolic self-organization, which converts nutrients into energy and sustains organ function; (ii) immune defense, which identifies and clears pathogens and abnormal cells; (iii) self-healing, which accomplishes regeneration and repair after injury; (iv) wear resistance, which repairs DNA, removes damaged proteins and maintains mitochondrial health; and (v) adaptability, which accommodates internal and external change through the microbiota, organ compensation and cellular reprogramming [4,6].

## 3. Head Goose Molecules and the “HGM–Neg-Entropy–Drug Cloud” Axis

Collective behavior in nature frequently depends on a leading individual that coordinates the activity of the group; this hierarchical organization has a direct analog in the cell. Drawing on this analogy and on the neg-entropy view of medicine first formalized in Engineering [4] and extended in Acta Pharmaceutica Sinica B [7], academician Jian-Dong Jiang proposed the concept of HGMs and articulated an integrated “HGM–neg-entropy–drug cloud” framework for the holistic treatment of chronic complex disease [7,8].

Unlike a conventional target, an HGM exerts an activating form of regulation that operates through the body’s own networks, thereby avoiding the toxicity and compensatory resistance that arise when a single pathway is forcibly over-blocked [8]. Pharmacologically, an HGM displays a characteristic set of properties: it is the upstream core node engaged directly by the drug; experimental perturbation of its expression abolishes the drug’s integrated effect; and it simultaneously recruits several intrinsic neg-entropy systems to generate a downstream drug cloud. Table 1 contrasts the network-regulatory and pharmacological features of conventional pathway targets with those of HGMs.

The regulatory axis mediated by an HGM follows a consistent temporal sequence. In disease, the HGM’s constitutive control is suppressed, the intrinsic neg-entropy systems become inactive, and the organism enters an entropy-driven pathological state. A drug then specifically activates the HGM, reopening the top-level regulatory switch. Cascade signaling simultaneously initiates multiple intrinsic neg-entropy repair pathways and drives coordinated, multi-organ reconstruction of physiological order. Finally, through the drug cloud network, metabolic, immune and self-healing systems become synchronized, systemic biological entropy falls, and the vicious cycle of pathological homeostasis is reversed [8].

## 4. The Collection at a Glance

The seven contributions to this edition are summarized in Table 2 and discussed below. Although they address distinct diseases, several share a common mechanistic logic: remodeling of metabolism or the microbiota, activation of coordinated multi-pathway responses, and restoration of homeostasis rather than simple suppression of a marker.

Respiratory disease is represented by the study of chlorogenic acid (CGA) in experimental asthma. Zhou and colleagues show that CGA attenuates airway inflammation, lowers Th2 cytokines (IL-4, IL-5) and IgE, and ameliorates lung pathology. Integrated lipidomics reveals that asthma is associated with disordered docosahexaenoic acid (DHA) metabolism and an accumulation of pro-ferroptotic lipid-peroxidation products (e.g., 11-HDoHE, 14-HDoHE), a profile reversed by CGA. Mechanistically, CGA activates the Nrf2 antioxidant pathway, upregulating the ferroptosis-defense genes *SLC7A11* and *GPX4* and thereby suppressing iron-dependent lipid-peroxidative death. The study links a dietary polyphenol to the coordinated correction of lipid metabolism, redox defense and cell death—an order-restoring response rather than a single anti-inflammatory action.

Metabolic disease is addressed by two studies. Zhang and colleagues demonstrate that gastrodin, derived from *Gastrodia elata*, ameliorates type II diabetes through a YY1–FXR–bile acid axis. Gastrodin reduces hepatic expression of the transcription factor YY1; because YY1 negatively regulates the farnesoid X receptor (FXR), its reduction restores FXR signaling, which acts through SHP to inhibit CYP7A1, decrease hepatic chenodeoxycholic acid production and reshape secondary bile acid formation via enterohepatic circulation. These changes promote GLP-1 and insulin secretion and lower blood glucose, while gastrodin additionally improves lipid accumulation, inflammation, antioxidant capacity and gut microbial composition, and repairs liver and pancreas pathology. The work illustrates how a single natural compound can rebalance an enterohepatic metabolic network. The renal–infectious dimension of metabolic and inflammatory disease is explored by Zhao and colleagues using *Polygonum capitatum* in a rat model of acute pyelonephritis. Combining microdialysis-coupled HPLC-MS/MS pharmacokinetics, pharmacodynamics, PK–PD analysis and renal metabolomics, they identify gallic acid, protocatechuic acid, methyl gallate and quercitrin as candidate active constituents and show that treatment partially reverses disturbances in glycerophospholipid metabolism and the tryptophan–kynurenine pathway while exerting antibacterial and anti-inflammatory, renoprotective effects.

Microbiota-dependent efficacy is a central theme of the edition. Shen and colleagues report that the plant-based formulation Dengzhan shengmai (DZSM), used clinically for cerebrovascular disease, alleviates DSS-induced ulcerative colitis by remodeling the gut microbiota. DZSM enriches short-chain fatty acid (SCFA)-producing bacteria and elevates colonic SCFAs; butyrate, in turn, upregulates the sodium-dependent vitamin C transporter SVCT1 in colonic epithelial cells, enhancing cellular vitamin C uptake. Butyrate and vitamin C then synergize to exert antioxidant and anti-inflammatory effects and reinforce the epithelial barrier, with fecal microbiota transplantation confirming the microbiota-mediated mechanism. The study defines a novel butyrate–SVCT1–vitamin C axis and exemplifies the adaptability capacity of neg-entropic regulation. In a complementary brief report, Ding and colleagues show that berberine protects against *Shigella flexneri*-induced dysentery by upregulating the barrier proteins ZO-1, occludin and E-cadherin, attenuating M1 macrophage polarization, restoring the Th1/Th17/Treg balance (reducing IFN-γ and IL-17A while increasing IL-10 and TGF-β1), and increasing secretory IgA. Together, these two studies demonstrate how natural products recruit microbial and immune networks to restore barrier and immune homeostasis.

Cardiovascular disease is represented by the study of glycyrrhizic acid (GL), a triterpenoid glycoside from the “medicine food homology” herb *Glycyrrhiza glabra*, in high-fat-diet-fed ApoE−/− mice. Because orally administered GL is poorly absorbed, its anti-atherosclerotic action is microbiota-dependent, as confirmed by antibiotic depletion and fecal microbiota transplantation: GL reverses gut dysbiosis, repairs the intestinal barrier and lowers circulating lipopolysaccharide. Integrated metagenomics and metabolomics show that GL redirects microbial tryptophan metabolism toward indole-3-lactic acid (ILA). The ILA-activated aryl hydrocarbon receptor (AhR) then physically binds the NF-κB subunit p65 in the cytoplasm, preventing p65 nuclear translocation and suppressing the adhesion molecules VCAM1 and ICAM1, thereby reducing endothelial inflammation and plaque burden. The work defines a microbiota–vascular ILA–AhR–p65 axis and offers a clear example of network-level, homeostasis-restoring protection.

Finally, the review by Hu and colleagues examines the therapeutic potential of dihydroartemisinin (DHA), the active metabolite of the artemisinin family, in cancer. Beyond its established antimalarial role, DHA displays broad antitumor activity through multiple molecular mechanisms, and the review systematically evaluates strategies to improve its bioavailability and efficacy, its value in combination with conventional agents, and the challenges of clinical translation. As a multi-pathway, increasingly well-characterized natural agent, DHA illustrates both the promise and the methodological agenda of nature-based oncology.

## 5. Head Goose Molecules Across Four Disease Landscapes

To connect the empirical studies in this edition to the broader theoretical framework, it is instructive to examine four clinically validated small molecules that act through disease-specific HGMs across cardiovascular, metabolic, infectious–immune and oncological disease [7,8]. Their regulatory features are compared in Table 3.

In the cardiovascular landscape, higenamine targets a composite HGM of the sinoatrial node: the β2-adrenoceptor and the L-type Ca2+ channel, which jointly govern automaticity and maintain electrophysiological–metabolic homeostasis [9]. Higenamine preferentially accumulates at pacemaker-cell membranes, agonizes the β2 receptor to activate the cAMP cascade and concurrently increases the open probability of the L-type Ca2+ channel. Blocking either component abolishes the drug’s effects on automaticity, conduction and perfusion, with no compensatory pathway able to substitute—consistent with the irreplaceable, top-level character of an HGM. Downstream, three neg-entropy systems are engaged in a drug cloud: mitochondrial energy efficiency and coronary perfusion (energy neg-entropy), regularization of sinoatrial–atrioventricular conduction (electrophysiological neg-entropy), and correction of ischemic microcirculatory disorder (circulation neg-entropy).

In the metabolic landscape, berberine acts through mitochondrial SIRT3 [10]. It accumulates in hepatocyte mitochondria, binds and activates SIRT3, and thereby promotes deacetylation of the complex I subunit NDUFS1, producing a selective and reversible dissociation of mitochondrial complex I rather than a direct enzymatic blockade; in SIRT3-knockout animals, the glucose-lowering, lipid-lowering, anti-inflammatory and anti-steatotic effects of berberine are markedly attenuated [10]. From this hub, a drug cloud restores metabolic self-organization (AMPK activation, suppressed hepatic gluconeogenesis, enhanced fatty-acid oxidation and LDLR/PCSK9 regulation), self-healing anti-inflammatory pathways (suppression of NF-κB and NLRP3), and gut–liver adaptability (enrichment of beneficial bacteria and short-chain fatty acids), lowering systemic metabolic entropy. Berberine has accordingly been described as a neg-entropic modulator that recouples energy, immune and microbial circuits across organs.

In the infectious–immune landscape, azvudine acts through a thymic HGM [11,12]. It homes to the thymus and, within CD4 + CD8+ double-positive T cells, is phosphorylated through the Dck/Cmpk1 kinase hub to its active triphosphate; this hub improves double-positive T-cell survival, reduces aberrant apoptosis and drives differentiation toward mature single-positive thymocytes, which then release circulating keratinocyte growth factor as a cross-organ messenger that spreads immune repair to the periphery [12]. Simultaneously, azvudine triphosphate directly engages the viral RNA-dependent RNA polymerase as a substrate decoy, terminating pathogen replication and removing the external driver of immune disorder. Athymic models, which lack the double-positive T-cell compartment and the Dck/Cmpk1 axis, show markedly attenuated immune-repair and survival benefits. The combination of internal immune-network remodeling and external viral suppression realizes a treatment of both symptoms and root causes.

In the oncological landscape, CGA acts through a dual HGM axis [13,14]. Affinity-based target fishing identified mitochondrial ACAT1 as a principal direct target; CGA binds the ACAT1 tetramer and abolishes its activating Y407 phosphorylation without disrupting the tetramer–monomer equilibrium, reversing the Warburg shift and restoring oxidative phosphorylation [13,14]. In parallel, CGA elevates SUMO1 to promote c-Myc sumoylation and degradation, suppressing cancer-stem-cell stemness and de-differentiation [14]. The resulting drug cloud restores differentiation (upregulating the glioma markers GFAP and Tuj1), enforces error correction and anti-proliferation (p21-mediated arrest and inhibition of invasion), and rebuilds immune defense by downregulating PD-L1 and promoting CD8+ T-cell infiltration, converting an immune-cold microenvironment into a hot one. Validated principally in glioma, this differentiation-oriented, homeostasis-restoring mode differs fundamentally from nonselective cytotoxic chemotherapy.

## 6. Unifying Principles, Translational Bottlenecks and Outlook

Despite targeting different HGMs in different diseases, the molecules above converge on a set of unifying regulatory principles: a common initiation point at the top of the signaling network; an activating rather than blocking mode of intervention; a network-level drug cloud effect that engages multiple pathways rather than one; a favorable safety profile conferred by operating through the body’s own physiological networks; a consistent fit to the “HGM initiation → drug cloud signaling → neg-entropy homeostasis” framework; and a therapeutic goal defined as reconstruction of homeostasis rather than suppression of an individual marker [7,8]. These principles offer operational criteria by which candidate HGMs may be recognized.

Translation nonetheless faces substantial bottlenecks. A multi-effective natural small molecule may bind hundreds of proteins, and deconvolution of the primary, decisive target remains laborious in the absence of high-throughput, dedicated screening systems. Entropy measures such as Shannon and multi-scale entropy remain largely research tools, and no standardized clinical bio-entropy assay yet exists to serve as a routine pharmacodynamic marker. Many active constituents—berberine and CGA among them—exhibit limited oral bioavailability and weak tissue targeting, constraining in vivo HGM activation. Finally, several drugs depend on composite or dual HGMs whose interaction networks are incompletely mapped, complicating the rational design of artificial targeted molecules.

Addressing these challenges suggests a focused agenda. First, scale the discovery of natural-product-derived HGM agonists through systematic screening of medicinal plants combined with reverse target-tracing. Second, optimize tissue-targeting and bioavailability through structural modification and prodrug design, building on the mitochondrial homing of berberine and the thymic homing of azvudine. Third, design dual HGMs that engage two complementary hubs, extending the ACAT1/c-Myc precedent. Fourth, construct rational combinations in which an HGM-directed agent restores intrinsic neg-entropy while a conventional single-target drug rapidly contains acute injury, reducing resistance and improving overall efficacy. Fifth, establish standardized multi-scale bio-entropy models as quantitative endpoints for evaluating new therapies for chronic disease.

## 7. Conclusions

The second edition of this Special Issue demonstrates, across a diverse set of diseases, how natural products engage the body’s intrinsic regulatory networks. Read through the lens of HGMs, neg-entropy homeostasis and the drug cloud, these studies support a shift from blocking isolated pathogenic nodes toward activating the systems by which the organism restores its own order. The framework remains young, and its target-deconvolution, bioavailability and quantitative-entropy challenges are real; yet the consistent convergence of clinically validated molecules on a common regulatory logic is striking.

## Figures and Tables

**Table 1:** Network-regulatory and pharmacological features of conventional pathway targets versus head goose molecules.

Feature	Conventional Pathway Target	Head Goose Molecule
Position in network	Mid-/downstream node of a pathway	Top-level master hub of the network
Scope of regulation	Local effect within a single pathway	Global effect across cells and organs
Neg-entropy role	Relieves damage to a single neg-entropy capacity	Simultaneously activates multiple intrinsic neg-entropy systems
Pharmacological profile	Blocking, symptom-oriented, prone to compensatory resistance	Activating, root-cause-oriented, multi-effective and low-toxicity

**Table 2:** Summary of the contributions published in this Special Issue.

Type	Compound/Formulation	Disease or Model	Principal Mechanism/Axis
Research	Chlorogenic acid	Allergic asthma (OVA mouse)	Reprograms DHA metabolism; activates Nrf2–SLC7A11–GPX4 to suppress ferroptosis
Research	Gastrodin	Type II diabetes (mouse)	YY1–FXR–bile acid axis; restores enterohepatic bile acid signaling and GLP-1/insulin secretion
Research	*Polygonum capitatum* extract	Acute pyelonephritis (rat)	Integrated PK–metabolomics; modulates glycerophospholipid and tryptophan–kynurenine pathways
Research	Dengzhan shengmai	Ulcerative colitis (DSS mouse)	Microbiota-driven butyrate–SVCT1–vitamin C axis
Research	Glycyrrhizic acid	Atherosclerosis (ApoE−/− mouse)	Microbiota–vascular ILA–AhR–p65 axis; suppresses endothelial adhesion molecules
Review	Dihydroartemisinin	Cancer	Multi-mechanism antitumor actions; bioavailability and combination strategies
Brief report	Berberine	*Shigella*-induced dysentery (mouse)	Restores intestinal barrier and macrophage/T-cell balance; raises sIgA

**Table 3:** Representative small molecules acting through head goose molecules across four disease landscapes.

Molecule	Disease	Target Organ/Cell	Core Head Goose Molecule	Neg-Entropy Capacity Restored	Refs.
Higenamine	Bradyarrhythmia/sick sinus syndrome	Sinoatrial node; pacemaker cells	β2-adrenoceptor–L-type Ca2+ channel complex	Cardiac energy metabolism and electrophysiological order	[9]
Berberine	Metabolic syndrome (T2DM, dyslipidemia, MASLD)	Liver/gut; hepatocytes, enterocytes, skeletal muscle, adipose	Mitochondrial SIRT3 deacetylase	Glucose–lipid metabolism, anti-inflammation, antioxidant defense	[10]
Azvudine	Viral infection/immune decline	Thymus; CD4 + CD8+ double-positive T cells	Dck/Cmpk1 kinase complex; viral RdRp	Central immune (T-cell development) and anti-inflammatory defense	[11,12]
Chlorogenic acid	Glioma/solid tumors	Tumor tissue; glioma stem cells, tumor and immune cells	ACAT1 and c-Myc	Cell differentiation, metabolic reprogramming, antitumor immunity	[13,14]

## Data Availability

No new data were created or analyzed in this study. Data sharing is not applicable to this article.

## References

[1] Newman D.J., Cragg G.M. (2020). Natural Products as Sources of New Drugs over the Nearly Four Decades from 01/1981 to 09/2019. J. Nat. Prod..

[2] Hopkins A.L. (2008). Network Pharmacology: The Next Paradigm in Drug Discovery. Nat. Chem. Biol..

[3] Talevi A. (2015). Multi-Target Pharmacology: Possibilities and Limitations of the “Skeleton Key Approach” from a Medicinal Chemist Perspective. Front. Pharmacol..

[4] Gao T.-L., Guo H.-H., Jiang J.-D. (2024). Neg-Entropy Mechanism as a Target for Natural Medicines. Engineering.

[5] Wang Z. (2022). The Entropy Perspective on Human Illness and Aging. Engineering.

[6] Jiang J.-D. (2024). Editorial for the Special Issue on Natural Medicine. Engineering.

[7] Li R., Gao T.-L., Ren G., Wang L.-L., Jiang J.-D. (2026). Neg-Entropy Is the True Drug Target for Chronic Diseases. Acta Pharm. Sin. B.

[8] Li R., Ren G., Han Y.-X., Wang L.-L., Jiang J.-D. (2024). Searching for Head Goose Molecules as Drug Targets. Chin. Med. Biotechnol..

[9] Chen D.-T., Rao W., Shen X., Chen L., Wan Z.-J., Sheng X.-P., Fan T.-Y. (2022). Pharmacological Effects of Higenamine Based on Signalling Pathways and Mechanism of Action. Front. Pharmacol..

[10] Wang X., Li H., Li J., Lei L., Xu J., Sun H., Li J., Jiang J., Li H., Tang M. (2025). Berberine Dissociates Mitochondrial Complex I by SIRT3-Dependent Deacetylation of NDUFS1 to Improve Hepatocellular Glucose and Lipid Metabolism. Sci. China Life Sci..

[11] Zhang J.-L., Li Y.-H., Wang L.-L., Liu H.-Q., Lu S.-Y., Liu Y., Li K., Liu B., Li S.-Y., Shao F.-M. (2021). Azvudine Is a Thymus-Homing Anti-SARS-CoV-2 Drug Effective in Treating COVID-19 Patients. Signal Transduct. Target. Ther..

[12] Sheng N., Li R., Li Y., Wang Z., Wang L., Li Y., Zhang J., Jiang J. (2024). Selectively T Cell Phosphorylation Activation of Azvudine in the Thymus Tissue with Immune Protection Effect. Acta Pharm. Sin. B.

[13] Wang Q., Du T., Zhang Z., Zhang Q., Zhang J., Li W., Jiang J.-D., Chen X., Hu H.-Y. (2024). Target Fishing and Mechanistic Insights of the Natural Anticancer Drug Candidate Chlorogenic Acid. Acta Pharm. Sin. B.

[14] Huang S., Wang L.-L., Xue N.-N., Li C., Guo H.-H., Ren T.-K., Zhan Y., Li W.-B., Zhang J., Chen X.-G. (2019). Chlorogenic Acid Effectively Treats Cancers Through Induction of Cancer Cell Differentiation. Theranostics.

